# Rethinking Zinc—Do We Need Catch-Up Evidence or Just Catch-Up Care?

**DOI:** 10.1016/j.advnut.2024.100320

**Published:** 2024-10-16

**Authors:** Stephanie P Gilley, Nancy F Krebs

**Affiliations:** Section of Nutrition, Department of Pediatrics, University of Colorado Anschutz Medical Campus, Aurora, CO, United States

Zinc is an essential trace mineral with myriad functions in the human body. It is a component of thousands of metalloproteins and zinc-binding proteins, often as an active part of the catalytic site. Zinc participates in a wide range of biologic functions, with major roles in nucleic acid metabolism, gene transcription, neuronal signal transduction, cellular proliferation, cell lineage differentiation and migration, and apoptosis regulation [1,2]. The diversity and complexity of zinc’s functions highlight its importance in health while also complicating its assessment, and thus its consideration, in clinical practice.

In this issue of *Advances in Nutrition*, Consales et al. [3] expertly summarize the current knowledge on the role of zinc in the health of premature infants. Their comprehensive review addresses the relationship of other nutrients to zinc absorption and highlights the benefits and limitations of available indices of zinc status. Zinc deficiency is increasingly recognized as a contributor to morbidity and mortality in premature infants [4]. The strongest evidence of zinc’s benefits remains its positive effects on both weight gain and linear growth. Multiple meta-analyses have shown improved weight, length, and head circumference z-scores in premature infants who received zinc supplementation [4,5]. The positive effects on growth appear to persist until at least 3 to 6 mo of corrected gestational age, although the evidence is more sparse [6].

Despite this recognition, attention to zinc in the premature population has lagged behind that of several other nutrients. In low-resource settings and the global health field, substantial effort has been made toward mitigating mild-to-moderate zinc deficiency [7]. However, in higher resource settings in which zinc deficiency in the general population is rare, the clinical relevance of this micronutrient has received less consideration. Traditionally, in the care of preterm infants, assessments of micronutrients such as iron, calcium, phosphorus, and vitamin D are more closely monitored and routinely supplemented. Like many other nutrients with great diversity of functions, such as proteins, there are no body stores of zinc, and it lacks a singular biomarker that accurately reflects whole body zinc “status” [1,7].

Despite longstanding knowledge of the importance of zinc, deficiency in premature infants remains underrecognized, and published reports of severe zinc deficiency in preterm infants are still readily found [8]. We suggest this situation is due to several reasons. First, zinc requirements for premature infants are still an area of active debate. The most recent expert guidelines suggest higher relative needs for the smallest infants, with up to 3 mg/kg/d recommended for infants weighing <1000 g [9]. Second, donor human milk contains variable but generally substantially lower zinc concentrations than the milk of a premature infant’s mother, partly due to processing but also due to its origins from mothers who are many months postpartum when milk zinc concentrations have markedly declined from that of early postpartum period [10]. Although zinc concentrations in preterm and term human milk differ based on postmenstrual age, they are equivalent at the same postpartum age and demonstrate similar physiologic decline [11]. Third, the majority of feeding regimens, including donor or maternal milk with human milk fortifier and premature infant formulas, provide intakes of ∼2 mg/kg/d, although the actual amounts vary considerably, which may be insufficient to meet the estimated needs of the most vulnerable infants, especially those with immature and/or impaired gastrointestinal or renal function [2,9]. Finally, clinical symptoms of mild-to-moderate deficiency are often nonspecific and can be easily missed or attributed to other causes, particularly in the absence of a reliable biomarker. Appearance of the classic dermatitis indicates progression to severe deficiency.

Several key knowledge gaps remain. Existing studies are small in size relative to the wide range of potential therapeutic interventions and clinical course of preterm infants, vary greatly in zinc dosing, and sometimes have had contradictory results [4]. There is an essential need for large, multicenter trials strategically designed to test the ideal amount, form, and timing of zinc administration in premature infants. Studies would ideally report functional outcomes, particularly growth outcomes including body composition, in addition to mortality, sepsis, necrotizing enterocolitis, vomiting, diarrhea, and a measure of neurodevelopment [[4], [5], [6]]. Endpoints would also usefully extend beyond neonatal intensive care unit discharge to more comprehensively understand the long-term effects of zinc supplementation [6]. Such studies should also place a special focus on the earliest gestations (e.g., <27 wk) with a plan to address the needs of the most vulnerable infants at the lowest limits of viability.

However, results from meta-analyses published in the last 3 y [[4], [5], [6]] suggest that sufficient evidence may already be available to support more routine zinc supplementation in preterm infants. Clinicians can be reassured that empiric zinc supplementation has been associated with minimal risk. Studies co-reporting copper levels have not found evidence for hypocupremia [4,6,12]. Vomiting, the most common side effect of higher dose (10–20 mg/d) zinc supplementation for the treatment of diarrhea for young children in low-resource settings, has not been reported in premature infants, although few studies specifically mention this potential adverse effect [4,5]. Therefore, a low threshold for additional zinc supplementation is warranted, especially for infants with suboptimal growth or conditions that increase the risk of deficiencies such as extreme prematurity, bowel surgery, diarrhea, enteral losses, or dialysis. Lastly, zinc supplementation should be considered when planning for discharge of any infant receiving human milk, as the current liquid infant multivitamins contain no or negligible zinc [8].

Decades of accumulated evidence demonstrate the critical role of zinc for infants born prematurely. Yet, best clinical practices remain unclear, especially as needed to keep pace with the remarkable advances in the care and survival of the most vulnerable neonates. Progress will most satisfactorily be achieved with rigorously conducted, adequately powered randomized controlled trials focused on clinical outcomes.

## Conflict of interest

SPG reports a relationship with Bobbie Labs that includes grant funding. The other author reports no conflicts of interest.

## Funding

The authors reported no funding received for this study.
